# Peptide-Based Immunotherapeutics and Vaccines 2017

**DOI:** 10.1155/2018/4568239

**Published:** 2018-07-15

**Authors:** Pedro Reche, Darren R. Flower, Masha Fridkis-Hareli, Yoshihiko Hoshino

**Affiliations:** ^1^Department of Immunology & O2, Facultad de Medicina, Universidad Complutense, 28040 Madrid, Spain; ^2^School of Life and Health Sciences, Aston University, Birmingham B4 7ET, UK; ^3^ATR, LLC, Worcester, MA 01606, USA; ^4^Department of Mycobacteriology, National Institute of Infectious Diseases, Higashi-Murayama, Tokyo 189-0002, Japan

The increasing understanding of the immune system and the critical role of antigenic epitopes in eliciting robust immune responses has led to the development of peptide vaccines. Peptide-based vaccines or—more technical—epitope ensemble vaccines represent an alternative approach to the discovery of disease-specific prophylactic and therapeutic vaccines, distinct from other vaccine moieties, such as attenuated or killed whole pathogen vaccines, subunit or toxoid vaccines, and carbohydrate-based vaccines. Epitopes represent the relevant part of the antigen recognized by T and/or B cells, mediating adaptive immunity. Consequently, the major potential of epitope vaccine ensembles is that of inducing desirable T and B cell-mediated immune responses. The risk of causing pathogenic or off-target responses with epitope vaccines is thus much lower than with convectional vaccines and can, on that basis, be regarded as safer. Epitope ensemble vaccines are also very versatile and can be formulated as synthetic peptides or encoded as DNA and RNA formulations. Currently, there are many epitope vaccines under development, spanning a wide range of diseases, including chronic viral infections and therapeutic anticancer vaccines, but as yet none are available. The case for epitope ensemble vaccines is nonetheless compelling, and the research community remains certain it is only time that separates us from a viable and deployable vaccine.

Developing a successful peptide-based vaccine involves identifying disease-specific epitopes inducing protective immunity and tackling a number of steps, such as determining appropriate means of epitope delivery and overcoming the intrinsically low immunogenicity of isolated epitopes. Following the success of previous special issues on peptide-based vaccines and immunotherapeutics, we present a new edition of the special issue in which we have incorporated seven original articles and two reviews, addressing various aspects of peptide-based vaccine design.

The reviews in the issue deal with epitope databases and epitope prediction. Peptide databases with information on T and B cell epitopes and peptide binding to MHC molecules are important tools for analyzing immune responses, benchmarking predictive methods, generating new ones, and developing peptide-based immunotherapeutics [1]. Currently, there are a number of online resources providing this type of information [2] but the Immune Epitope Database (IEDB) is the largest and most comprehensive epitope database. Thus, in this issue, we are pleased to feature a review by W. Fleri et al., in which the authors describe how the data are entered and retrieved from IEDB. B and T cell epitopes can be predicted from the relevant antigens with the help of bioinformatics tools. In this issue, J. L. Sanchez-Trincado et al. analyzed aspects of antigen recognition by T and B cells that are relevant for epitope prediction and provided a systematic and inclusive review of available tools, paying particular attention to their foundations. In the review, the authors also provide arguments on why B cell epitope prediction is less accurate and practical than T cell prediction and introduce solutions to solve some of the problems associated with epitope-vaccine design.

The original articles in this special issue include two papers focusing on computational vaccine design. In one, A. R. Oany et al. proposed a peptide vaccine candidate for shigellosis consisting of four predicted cytotoxic T cell (CTL) epitopes from the SigA antigen, which is known to be highly immunogenic. These four candidates are conserved among *Shigella* species and provide wide population coverage. In addition, the authors identified that MHC II molecules could also present two of the CTL epitopes and thus stimulate T helper responses. In the other, J. Alonso-Padilla et al. have expanded on an emerging strategy that relies on the use of experimentally defined T-cell epitopes [3] and formulated a prophylactic epitope vaccine against EBV infection that includes both T and B cell epitopes. The T cell component consists of experimentally defined CD8 and CD4 T cell epitopes from various EBV antigens that are conserved and can be presented by a large number of human MHC molecules, while the B cell component includes experimental B cell epitopes mapping on the ectodomain of EBV envelope proteins and exhibiting a high degree of flexibility and solvent accessibility.

The experimental articles in the issue range widely. We have a work by M. Niki et al. which aims to identify appropriate antigens for tuberculosis (TB) vaccines. The study is an extension of a previous work in which the authors did a cross-sectional assay in TB patients [4]. Here, the authors used a different cohort and did a longitudinal assay, finding immunoglobulin responses to antigens that correlated to several clinical parameters. These results provide insights into the development of a novel TB vaccine inducing protective humoral immunity.

Dendritic cell- (DC) and peptide-based immunotherapies often go hand in hand. In this issue, J. Lo et al. investigate on whether DCs unpulsed or pulsed with antigenic dominant determinants (DD), subdominant determinants (SD), and ignored determinants (ID) could prevent type 1 diabetes (T1D) in a mouse model. The authors found that diabetes was significantly delayed by DCs pulsed with SD or ID peptides. Moreover, they also found that Tregs from DC-treated mice proliferated more actively and showed enhanced immunosuppressive activities. Overall, this study demonstrates that DC therapy leads to long-lasting immunomodulatory effects in an antigen-dependent manner, providing support for DC-guided peptide-based interventions for autoimmune diabetes.

Toxicity is a handicap for vaccine design and, here, A. Latanova et al. present a work addressing this issue. In the study, the authors fused a flaviviral leader peptide to reverse transcriptase (RT), a crucial target of immunotherapy against drug-resistant HIV-1. This fusion allowed RT secretion and reduced its toxicity and ability to induce oxidative stress, with no major effects on its immunogenicity. Subsequently, the authors proposed the use of leader peptides to increase safety of RT-based DNA vaccines. Genetic diversity of pathogens remain also a substantial obstacle for vaccine design. V. S. Kichatova et al. addressed this subject for human hepatitis C virus (HCV). These authors characterized the occurrence of IFN resistance-conferring mutations in HCV isolates circulating in the Russian Federation, identifying that the spread of viral variants was linked to mutations on HCV-specific CTL epitopes in association with the immunogenetic background of HCV-infected individuals. These results are useful in identifying those individuals in need of IFN-free treatments and for developing epitope-based vaccines that circumvent viral immune escape.

Finally, we include a work by I. Soria et al. showing that combining viral-specific B and T cell epitopes onto appropriated structures can increase immunogenicity and enhance protection. These authors work with foot-and-mouth disease virus (FMDV), which has a high morbidity in cloven-hoofed animals, like cattle and swine. In cattle, there are known FMDV-specific B and T cell epitopes that could be used for safer and more effective vaccines. However, immunization with linear synthetic peptides encompassing the epitopes has failed to induce protection in cattle. In contrast, in the study, the authors immunized cattle with a dendrimeric peptide structures consisting of 4 copies of a peptide encompassing a B cell epitope linked through thioether bonds to a single copy of a CD4 T cell epitope. As a result, I. Soria et al. obtained that these dendrimeric peptides elicited humoral and cellular immune responses that conferred partial protection against heterologous virus challenge.

In conclusion, the articles included in this special issue examined relevant aspects of peptide-based vaccines, and we trust that readers shall find them both interesting and motivating.

*Pedro Reche* *Darren R. Flower* *Masha Fridkis-Hareli* *Yoshihiko Hoshino*
